# Weevil x Insecticide: Does ‘Personality’ Matter?

**DOI:** 10.1371/journal.pone.0067283

**Published:** 2013-06-26

**Authors:** Juliana A. Morales, Danúbia G. Cardoso, Terezinha Maria C. Della Lucia, Raul Narciso C. Guedes

**Affiliations:** 1 Departamento de Entomologia, Universidade Federal de Viçosa, Viçosa, Minas Gerais, Brazil; 2 Departamento de Biologia Animal, Universidade Federal de Viçosa, Viçosa, Minas Gerais, Brazil; University of Plymouth, United Kingdom

## Abstract

An insect’s behavior is the expression of its integrated physiology in response to external and internal stimuli, turning insect behavior into a potential determinant of insecticide exposure. Behavioral traits may therefore influence insecticide efficacy against insects, compromising the validity of standard bioassays of insecticide activity, which are fundamentally based on lethality alone. By extension, insect ‘personality’ (i.e., an individual’s integrated set of behavioral tendencies that is inferred from multiple empirical measures) may also be an important determinant of insecticide exposure and activity. This has yet to be considered because the behavioral studies involving insects and insecticides focus on populations rather than on individuals. Even among studies of animal ‘personality’, the relative contributions of individual and population variation are usually neglected. Here, we assessed behavioral traits (within the categories: activity, boldness/shyness, and exploration/avoidance) of individuals from 15 populations of the maize weevil (*Sitophilus zeamais*), an important stored-grain pest with serious problems of insecticide resistance, and correlated the behavioral responses with the activity of the insecticide deltamethrin. This analysis was performed at both the population and individual levels. There was significant variation in weevil ‘personality’ among individuals and populations, but variation among individuals within populations accounted for most of the observed variation (92.57%). This result emphasizes the importance of individual variation in behavioral and ‘personality’ studies. When the behavioral traits assessed were correlated with median lethal time (LT_50_) at the population level and with the survival time under insecticide exposure, activity traits, particularly the distance walked, significantly increased survival time. Therefore, behavioral traits are important components of insecticide efficacy, and individual variation should be considered in such studies. This is so because population differences provided only crude approximation of the individual personality in a restrained experimental setting likely to restrict individual behavior favoring the transposition of the individual variation to the population.

## Introduction

“Curiosity killed the cat” is an animal aphorism that is widely used as a cautionary note to people who are too inquisitive, but the recent burgeoning interest in animal ‘personality’ most likely favors a more literal meaning of such statements. In fact, there has been a growing body of compelling evidence regarding the existence of ‘personality’ among animals and its eco-evolutionary importance [1]–[10]. Such growing interest naturally leads to conceptual misunderstandings, especially considering the long-standing interest and importance of human personality within the field of psychology and the more recent interest in animal ‘personality’ within biology, particularly in ecology and evolution [1], [2], [3], [5], [11]. Here, our working concept of ‘personality’ refers to an individual’s integrated set of behavioral tendencies inferred from multiple empirical measures, which is more commonly used in psychology than in animal biology, in which personality is frequently treated as a synonym of behavioral syndromes and related concepts [5], [11]. Furthermore, animal ‘personality’ as used in biology tend to focus on population variation rather than on individual variation [5], [7], [8], [11], and is prone to jingle-jangle-fallacies (i.e., same term referring to different concepts and different terms referring to the same concept) [11]. Our stated working concept circumvents both shortcomings.

The study of personality differences in humans has proven useful in predicting the manifestation of certain behaviors, such as job satisfaction, risk taking, and social stress reactions, among others [1], [12]–[15]. The expansion of personality studies to domestic animals led to improvements in animal production, welfare and conservation and thus drew attention from the general public [16]–[24]. By contrast, the study of animal ‘personality’ in arthropods lags behind, with studies of spiders and social insects as a main focus of attention [25]–[29], with very recent contributions regarding other species, such as the pea aphid *Acyrthosiphon pisum* and the confused flour beetle *Tribolium confusum* [30]–[33].

Theoretical considerations and frameworks attempting to address the roles of animal ‘personality’ variation within ecology and evolution have been receiving considerable attention, but adequate empirical tests of such hypotheses are lacking, especially those that consider testing the adaptive value of ‘personality’ [1]–[9]. In psychology, for instance, child personality has been associated with unintentional injury risk, and more specifically, with poison ingestion [12], [15], [34]–[37]. In applied entomology, the likelihood of insecticide exposure is potentially associated with insect behavioral traits. In fact, pesticide resistance mediated by insect behavior (i.e., avoidance) has been documented elsewhere [38]–[49]. However, the potential influence of ‘personality’ on insect mortality by insecticides has adaptive and applied consequences that have been neglected.

Bioassays of insecticidal activity usually consider mortality to be the assessment endpoint, particularly when insect pest species are considered, although insect behavior is an important determinant of insecticide exposure and, consequently, of its activity [42], [43], [50], [51]. There is no denying the importance of studying the lethal effects of insecticides; nevertheless, it is unwise to neglect the influence of insect behavior on insecticide efficacy, or for that matter, insect ‘personality’. Even when insect behavior is considered in insecticide studies, almost exclusively only the variation among insect populations is considered, as opposed to the variation within populations (i.e., individual variation), thus neglecting the importance of the latter [38]–[52]. Among studies of animal ‘personality’, the relative contribution of individual and population variation is also usually overlooked, as well as the multidimensional nature of ‘personality’. By contrast, the tendency has been to focus on a category of behavior and to explore its correlations across different contexts, commonly referred to as ‘behavioral syndromes’, which is a term that is frequently used as a synonym of ‘personality’ in animal studies [2], [4]–[11].

In this study, we assessed behavioral traits (within the following categories: activity, boldness/shyness, and exploration/avoidance) of the maize weevil *Sitophilus zeamais* Motschulsky (Coleoptera: Curculionidae) from 15 populations. The behavioral responses were tested for correlations with the efficacy of the pyrethroid insecticide deltamethrin. This analysis was performed at both the population and individual levels to determine the relative contribution of individual and population variation in the make-up of the insect ‘personality’, if indeed it exists, and to determine how individual and population variation in behavioral traits may relate to survival to insecticide exposure. As both behavior and insecticide susceptibility are conceptually attributes of the individual, we believe that individual variations in behavioral traits are fundamental contributors to ‘personality’ and its consequences, even if such ‘personality’ is expressed in a given population. However, insect ‘personality’, with its complexities, may not be necessary or even significant to explain insecticide susceptibility, which we also attempted to test in the present study.

## Materials and Methods

### Ethics Statement

No specific permits were required for the described studies, which were performed in the laboratory. Each of the insect colonies was initially established from over 200 individuals collected from storage units and maintained under mass rearing conditions at the Federal University of Viçosa. The insect species used here is a cosmopolitan pest species of cereal grains and is very common in Neotropical America and Africa. The study therefore did not involve any endangered or protected species.

### Insect Populations

Fourteen populations of the maize weevil that were collected from storage units across Brazil, and one from Paraguay, were used in the experiments. These populations were mostly collected within the last five years, except the populations from Jacarezinho and Juiz de Fora, which were collected in the late 1980s and late 1990s, respectively. These populations varied in their resistance to pyrethroid insecticides, with some susceptible populations (e.g., Sete Lagoas) and some insecticide-resistant ones (e.g., Jacarezinho and Juiz de Fora). The prevailing resistance mechanism is altered target site sensitivity with secondary involvement of enhanced detoxification by glutathione-*S-*tranferases and esterases [52]–[55]. The specimens of each population were reared on maize kernels free of insecticide residues in 1.5 L glass jars maintained under controlled environmental conditions of 27±2°C, 70±10% relative humidity and a 12 h photoperiod.

### Behavioral Bioassays

Adult sexed insects between one and three days old were used for both the behavioral and the insecticide bioassays, which were sequentially performed during one week with the same individuals. The behavioral bioassays were subjected to retest in subsequent days to ascertain the relative stability of the behavioral responses obtained before the (final) insecticide bioassay. The individual emerging insects were isolated, sexed using their pattern of rostrum texture and punctuation [56], and maintained in 20 mL transparent plastic vials containing maize kernels. Twenty-one individuals of each sex and from each population were subjected to the bioassays. The behavioral bioassays were performed under controlled laboratory conditions, as previously described for rearing insect populations, between 9∶00 am and 6∶00 pm. Six behavioral bioassays were performed focusing on measurable behavioral (or performance) traits demonstrating an individual’s ability to perform a task, exploring three of the five categories of behavioral propensity (or personality traits) proposed by Reále et al. [5]: activity, boldness/shyness, and exploration/avoidance. Insect activity was assessed through a walking bioassay, two flight bioassays (flight mill and free-fall), and a body righting bioassay. Boldness/shyness was assessed through a death-feigning bioassay, and exploration/avoidance was assessed through bioassays of intra- and interspecific interactions using maize kernels infested with adults of either the maize weevil (intraspecific interaction) or the lesser grain borer (*Rhyzopertha dominica*) (interspecific interaction).

#### Walking activity bioassay

Walking activity was recorded for 10 min in Petri dish arenas (9 cm diameter) whose inner walls were coated with Teflon PTFE (DuPont, Wilmington, DE, USA) to prevent insect escape, as described elsewhere [46]–[49], [57]. A single insect was released in the center of the arena, and its movement was recorded and digitally transferred to a computer using an automated video tracking system equipped with a CCD camera (ViewPoint Life Sciences, Montreal, Canada). The following characteristics were evaluated: distance walked (cm), walking velocity (cm/s), and resting time (s).

#### Free-fall flight bioassay

A hand-made square box (44 cm wide, 44 cm deep, 88 cm high) was used for the free-fall flight bioassay. The frame was made of wood, and the top was covered with thin transparent plastic film with a hole 5 cm in diameter located in the center, whereas the lateral sides were covered by organza tissue. The box was placed on a sheet of paper marked with a series of concentric circles 3 cm apart from one another. Each individual adult weevil was dropped through the central hole in the top of the box, and the landing distance from the center after wing fluttering was recorded following the methods adapted from a study on the cowpea beetle *Callosobruchus chinensis* [58]. The test was replicated three times, and the mean score was used as the flying activity.

#### Flight-mill bioassay

The flight-mill bioassay method used was that described by Riley et al. [59]. Each individual insect had its thorax attached by a thread to a rod in the mill, and the insect movement was recorded for 10 min, registering the wing beat and the number of turns of each insect in the mill, allowing us to estimate the distance flown (m).

#### Body righting bioassay

Each insect was placed on its dorsum in an arena, and the time that was taken to recover its regular ventral posture was recorded. The procedure was replicated three times, and the mean score was used as the body righting activity, adapting a bioassay developed for the red flour beetle (*Tribolium castaneum*) [60].

#### Death-feigning bioassay

Death-feigning was induced by dorsally prodding the adult insect with a fine-haired brush and subsequently recording the time taken for the insect to start moving after reaching its characteristic death-feigning (or thanatosis) posture. This posture in the maize weevil involves leg contraction and subsequent immobilization upon prodding. The procedure was replicated three times, and the mean score was used as the duration of death-feigning behavior, again adapting a method developed for the red flour beetle [60].

#### Intra- and interspecific interaction bioassays

The exploration/avoidance behavioral category was assessed through bioassays of intra- and interspecific interactions using maize kernels infested by adults. A Petri dish arena (18 cm diameter) whose inner walls were coated with Teflon PTFE to prevent insect escape was used in this bioassay. The bottom of the arena was covered with millimeter paper on which the center was marked. A maize kernel infested with either a conspecific (for intraspecific interaction) or heterospecific (lesser grain borer *Rhyzopertha dominica*; for interspecific interaction) adult was placed at the center of the arena, and the weevil under investigation was placed at the edge of the arena. The weevil movement was recorded for 15 min, as well as the time (min) taken for the weevil to reach the infested kernel.

### Insecticide Survival Bioassays

The pyrethroid insecticide deltamethrin (K-Obiol 25EC, Bayer Crop Science, São Paulo, Brazil) was used at its registered label rate for maize weevil control in Brazil (0.5 ppm) [61]. One milliliter of insecticide emulsion was sprayed on 500 g of maize kernels placed in a rotary stainless steel container for homogenizing the grain during the application and until the grain was dry (one hour later). An artist’s air brush (Sagyma SW440A, Yamar Brasil, São Paulo, Brazil) coupled with an air pump (Prismatec 131A Tipo 2VC, Itu, SP, Brazil) was used for insecticide spraying, which was performed at a pressure of 0.7 kgf/cm^2^.

Transparent glass vials (25 mL) containing 15 g of maize kernels (sprayed with the deltamethrin formulation) were used as experimental units and received one insect each. Parallel bioassays were performed with insects of similar age (one week old) from the same sex, and populations (although not subjected to the behavioral bioassays previously described) exposed to maize kernels that were sprayed with only water acted as controls to detect any problems of high natural mortality compromising the insecticide bioassay, which was not the case. The cap area of the upper portion of the vials had its inner walls coated with Teflon PTFE, and the vial opening was covered with organza tissue tightly attached with a rubber band to prevent insect escape and allow gas exchange. Insect survival was assessed hourly during the 1^st^ 12 h and then at four hour intervals until 30 days after spraying. After 30 days, insect survival was recorded daily until each insect’s eventual death.

### Statistical Analyses

The results of the behavioral bioassays and their repetition (i.e., retesting) for each individual insect were subjected to correlation analysis to assess their consistency (PROC CORR in SAS (SAS Institute, Cary, NC, USA)) [62]. The behavioral traits assessed were subject to two sets of analyses, one focusing on the population (or inter-population) variation and the other focusing on individual (or within population) variation. Multivariate analysis of variance (complemented by univariate analysis of variance) and canonical variate analysis (CVA) were performed using population and sex as independent variables and the behavioral traits as dependent (response) variables, focusing on the population level variation using the CANDISC procedure in SAS [62]. For individual variation, the behavioral traits of individuals from both sexes and different populations were subjected to principal R-factor analysis using the FACTOR procedure in SAS with orthogonal (Varimax) rotation to reduce potential bias towards the first general factor [62]. Sampling adequacy was estimated using Kaiser’s measure for the purpose, which should significantly exceed 0.50 if the correlation matrix generated were suitable [62]. Factor analysis was preferred instead of the usual principal component analysis because the former aims to explain the variation in the measured variables by constructing latent ones (i.e., factors), enabling comparisons and generalizations across bioassays instead of solely focusing on data reduction [11], [63]. The relative contribution of populations and individuals within populations for the observed behavioral data variation was calculated using the modified analysis of variance proposed by Excoffier et al. [64].

The results of the survival bioassays were subjected to survival analysis using the non-parametric LIFETEST procedure in SAS [62], in which survival curves are obtained using Kaplan-Meyer estimators, allowing estimates of median survival time (LT_50_) for each sex and each population. These estimates were subsequently used as dependent (response) variables in multiple regression analysis using either canonical variates or behavioral data responses as independent variables with the GLM procedure in SAS and both ‘stepwise’ and ‘backward’ selection statements. Initially, only the original variables were used to construct the model, and subsequently, their improvement was attempted considering the interactions between the main original variables [62]. This model-building approach was used for exploring the population variation data, but for the individual variation data, the survival time (days) was modeled instead of the LT_50_, and the respective individual data, either from the main factors or behavioral data, were used for constructing multiple regression models with the GLM procedure in SAS and the ‘stepwise’ and ‘backward’ selection statements [62]. The assumptions of normality and homoscedasticity were evaluated before data analysis (UNIVARIATE procedure in SAS) [62], and wing beat and distance flown required transformation (log x+1) for the intended analyses.

## Results

### Repeatability and Multidimensional Behavioral Constructs

The results of testing and retesting the behavioral bioassays provided significant results when correlated with one another (n = 630, *p*<0.001), exhibiting correlation coefficients (r) ranging from 0.28 to 0.57 (Table 1). The multivariate analysis of variance performed indicated significant overall effects of population, sex and interaction of sex-population among the behavioral traits analyzed (Wilks’ lambda <0.94, F >3.34, *p*<0.001) (Table 1). Subsequent univariate analyses of variance performed for each behavioral trait assessed indicated that all behavioral traits were significantly affected by population, sex and/or their interaction (F_29,600_ = 1.93, *p*<0.003), except latency for interacting with a conspecific (F_29,600_ = 1.47, *p* = 0.06) (Tables 1 and 2). These results indicate great overall variation among populations and sex, which is also true for each individual behavioral trait assessed, except latency of conspecific interaction.

**Table 1:** Summary results of the univariate analyses of variance (ANOVA) and repeatability (test-retest) results of the behavioral traits of both sexes from 15 populations of the maize weevil (*Sitophilus zeamais*).

Sources of variation (ANOVA)	Degrees of freedom	Walking activity						Flight activity						Conspecific interaction latency (min)		Heterospecific interaction latency (min)		Duration of death-feigning (s)		Length of time to body righting (s)	
		Distance walked (cm)		Resting time (s)		Walking velocity (cm/s)		Horizontal dislocation upon fall (cm)		Wing beat (no.)		Distance flown (m)									
		F	*P*	F	*P*	F	*P*	F	*P*	F	*P*	F	*P*	F	*P*	F	*P*	F	*P*	F	*P*
Model	29	3.78	<0.001	2.07	<0.001	2.71	<0.001	2.68	<0.001	3.47	<0.001	2.73	<0.001	1.47	0.06	1.93	0.002	2.50	<0.001	5.46	<0.001
Error	600	–	–	–	–	–	–	–	–	–	–	–	–	–	–	–	–	–	–	–	–
Population	14	4.78	<0.001	2.86	<0.001	3.94	<0.001	1.97	0.02	4.47	<0.001	3.00	<0.001	1.49	0.11	3.08	<0.001	4.11	<0.001	10.23	<0.001
Sex	1	0.79	0.37	0.54	0.46	3.08	0.08	0.08	0.77	6.76	0.009	10.76	0.001	1.56	0.21	1.62	0.20	0.99	0.32	0.01	0.93
Population x sex	14	2.99	<0.001	1.39	0.15	1.45	0.12	3.97	<0.001	2.24	0.006	1.88	0.02	1.45	0.13	0.79	0.68	0.99	0.46	1.09	0.36
Repeatability (test-retest) correlations [n = 630]	r	0.57		0.49		0.51		0.37		0.43		0.35		0.28		0.31		0.48		0.50	
	*P*	<0.001		<0.001		<0.001		<0.001		<0.001		<0.001		<0.001		<0.001		<0.001		<0.001	

**Table 2:** Behavioral traits (± EPM) of both sexes from 15 populations of the maize weevil (*Sitophilus zeamais*).

Population	Sex	Walking activity			Flight activity			Conspecific interaction latency (min)	Heterospecific interactionlatency (min)	Duration of death-feigning (s)	Length of time to body righting (s)
		Distance walked (cm)	Resting time (s)	Walking velocity(cm/s)	Horizontal dislocation upon fall (cm)	Wingbeat (no.)	Distance flown (m)				
Abre Campo	Female	168.17±13.55	189.45±10.13	0.68±0.02	6.73±0.99	0.58±0.08	0.37±0.20	13.43±0.93	9.13±1.39	9.41±1.92	1.45±0.17
	Male	290.49±17.14	176.61±15.71	0.68±0.02	5.13±0.75	0.11±0.04	0.18±0.10	9.91±1.35	11.86±1.18	12.44±3.30	2.81±0.70
Amambay	Female	278.65±13.82	191.88±14.77	0.68±0.01	6.35±0.92	0.49±0.09	0.85±0.28	9.17±1.35	10.26±1.27	5.03±1.59	1.69±0.23
	Male	267.30±13.18	199.96±14.35	0.66±0.01	5.46±0.60	0.27±0.07	0.22±0.15	9.65±1.25	12.55±1.01	4.28±1.01	1.21±0.13
Esp. Sant. Pinhal	Female	253.69±11.37	189.54±12.03	0.61±0.01	4.67±0.28	0.34±0.09	0.34±0.19	7.93±1.16	9.79±1.15	10.73±3.47	4.69±1.16
	Male	265.72±12.91	181.21±12.82	0.63±0.01	5.11±0.36	0.31±0.08	0.25±0.17	8.03±1.26	11.39±1.05	18.77±7.45	4.76±1.67
Guarapuava	Female	253.42±11.44	213.77±13.15	0.65±0.01	4.98±0.72	0.22±0.07	0.22±0.15	6.14±1.15	5.67±1.40	10.89±2.94	1.85±0.39
	Male	246.84±15.49	216.02±14.86	0.64±0.02	4.38±0.23	0.31±0.07	0.00±0.00	9,79±1.25	5.48±1.15	7.38±1.28	2.04±0.45
Guaxupé	Female	300.56±12.79	174.22±10.37	0.71±0.02	5.89±0.58	0.22±0.02	0.09±0.04	9.21±0.11	9.90±1.28	5.47±1.06	0.99±0.09
	Male	251.87±10.39	208.99±0.01	0.64±0.21	4.90±0.15	0.61±0.09	0.82±0.29	9.54±1.36	12.69±1.07	4.91±1.15	0.96±0.15
Jacarezinho	Female	281.53±10.12	174.87±9.82	0.66±0.01	5.00±0.40	0.50±0.08	0.91±0.31	8.65±1.26	8.44±1.47	7.36±1.36	1.24±0.16
	Male	429.52±8.30	169.93±8.20	0.70±0.01	4.48±0.23	0.54±0.09	0.61±0.28	12.31±1.05	8.70±1.43	`7.71±1.95	1.69±0.30
Jacuí	Female	249.13±10.45	181.29±12.35	0.59±0.01	4.38±0.30	0.56±0.09	0.31±0.21	8.63±1.37	7.93±1.46	28.87±9.54	7.17±1.70
	Male	264.19±9.55	174.69±10.02	0.62±0.01	8.33±0.77	0.47±0.08	0.12±0.08	9.80±1.40	9.54±1.38	12.56±4.50	4.80±1.21
Juiz de Fora	Female	345.00±9.05	136.60±7.20	0.74±0.01	6.27±0.99	0.65±0.10	1.13±0.33	9.08±1.38	9.12±1.38	10.06±3.77	1.77±0.21
	Male	315.26±13.48	157.29±11.35	0.71±0.01	4.14±0.28	0.41±0.10	0.25±0.14	11.14±1.15	9.94±1.40	7.96±0.69	2.81±0.41
Piracicaba	Female	288.05±17.81	168.17±13.55	0.65±0.02	4.24±0.29	0.35±0.09	0.32±0.23	7.22±1.19	10.72±1.33	7.30±2.89	1.03±0.12
	Male	348.21±87.86	190.42±14.13	0.79±0.14	4.90±0.26	0.44±0.08	0.00±0.00	10.03±1.05	10.74±1.27	3.40±0.75	1.14±0.13
Proterito	Female	256.82±11.56	202.89±10.84	0.64±0.01	5.60±0.58	0.81±0.10	0.82±0.27	9.47±1.31	9.34±1.34	10.44±6.10	1.69±0.36
	Male	247.08±9.77	202.44±9.70	0.62±0.01	4.47±0.23	0.63±0.09	0.00±0.00	11.45±1.05	10.57±1.32	7.59±2.34	1.48±0.24
Rio Verde	Female	262.96±13.34	187.99±13.19	0.63±0.01	5.25±0.52	0.54±0.09	0.65±0.23	10.10±1.36	8.86±1.51	7.59±2.78	1.19±0.13
	Male	241.13±14.09	207.28±13.94	0.61±0.01	4.57±0.22	0.23±0.09	0.00±0.00	8.23±1.37	10.67±1.40	3.65±1.28	1.70±0.21
Sete Lagoas	Female	302.27±13.09	164.06±10.23	0.69±0.01	4.33±0.28	0.60±0.10	0.30±0.17	9.74±1.34	9.61±1.17	24.03±7.55	1.31±0.22
	Male	302.07±17.21	171.35±13.27	0.70±0.02	4.84±0.62	0.61±0.11	0.76±0.27	9.95±1.32	9.27±1.42	21.62±6.73	1.97±0.31
Unaí	Female	293.03±18.79	168.37±17.99	0.67±0.01	4.86±0.17	0.38±0.09	0.07±0.07	11.09±1.33	12.86±1.26	8.38±4.75	2.48±0.45
	Male	235.90±13.94	228.42±12.63	0.64±0.02	6.46±0.98	0.63±0.09	0.07±0.07	11.81±1.20	11.43±1.34	7.75±2.72	1.45±0.16
Viçosa	Female	268.94±13.64	174.51±13.27	0.64±0.02	5.24±0.23	0.39±0.09	0.21±0.15	10.98±1.30	12.10±1.05	7.48±1.74	2.10±0.30
	Male	311.85±13.22	149.02±11.11	0.69±0.01	5.71±0.86	0.19±0.06	0.37±0.20	8.53±1.14	10.27±1.31	7.02±1.27	2.13±0.34
Votuporanga	Female	270.52±15.26	187.43±13.81	0.65±0.02	4.81±0.32	0.47±0.09	0.98±0.23	10.10±1.33	9.78±1.27	9.16±3.45	2.97±0.45
	Male	291.70±12.75	190.58±10.53	0.71±0.03	4.92±0.29	0.49±0.08	0.69±0.25	9.75±1.08	7.37±1.21	14.44±2.66	2.39±0.26

**Figure 1:**
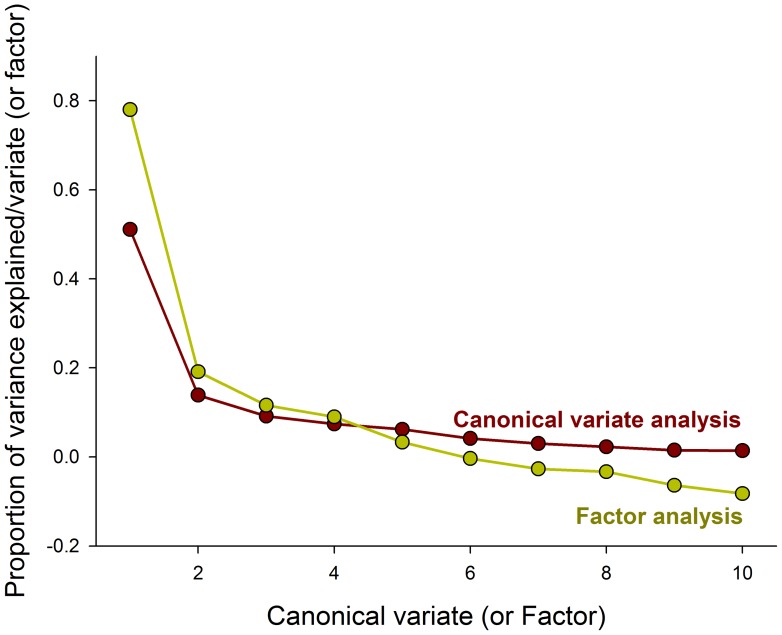
Proportion of the behavioral data variance explained by each canonical variate or factor generated from CVA or R-factor analysis, respectively.

**Figure 2:**
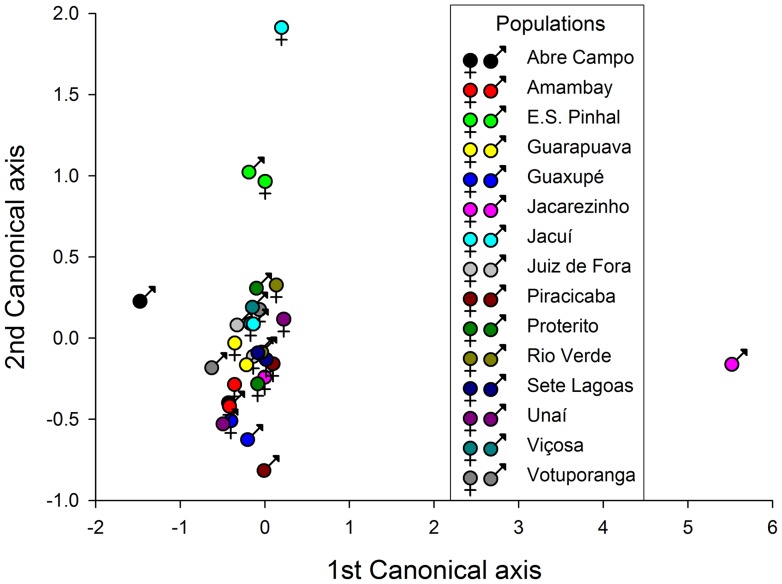
Ordination (CVA) diagram showing the behavioral divergence among males and females of populations of the maize weevil *Sitophilus zeamais* (see **Table 3**). The symbols are centroids of treatments representing the class mean canonical variates.

The multidimensional behavioral constructs representing the population-level ‘personality’ of each sex from each population were obtained with a CVA of the behavioral traits assessed. The CVA ordination generated six significant axes (*p*<0.05), with the first two explaining 64.75% of the observed variance (Fig. 1). The variable with greater canonical loadings accounting for most of the divergence among sex and populations was the distance walked (1^st^ canonical axis), followed by the duration of the death-feigning behavior and length of time to right the body (2^nd^ canonical axis), with an opposite contribution mainly from the walking velocity (Table 3). The ordination diagram derived from the CVA representing the prevailing ‘personality’ of each sex from each population emphasizes the distinction of males from the population of Jacarezinho (by the 1^st^ axis), mainly because of their extensive walking activity, and females from Jacuí (by the 2^nd^ axis), which exhibited delayed body righting and recovery from death-feigning (Fig. 2; Table 1).

**Table 3:** Canonical (for CVA) and factor (for principal R-factor analysis) loadings of the significant (*p*<0.05) canonical axes (CVA) and factors for the behavioral traits of both sexes from 15 populations of the maize weevil (*Sitophilus zeamais*).

Variables	Canonical axes						Principal main factors (orthogonally rotated)	
	1	2	3	4	5	6	1	2
Distance walked	**0.68**	−0.20	0.06	−0.15	**0.52**	0.06	**0.97**	−0.06
Resting time	−0.15	−0.23	−0.12	0.28	−**0.86**	−0.00	−**0.63**	−0.11
Walking velocity	0.09	−**0.49**	0.11	−0.16	**0.51**	0.11	**0.89**	−0.03
Horizontal dislocation upon fall	−0.20	0.02	0.11	**0.75**	0.28	−**0.48**	0.05	−0.23
Wing beat	0.17	−0.26	**0.83**	0.36	0.02	0.15	0.01	−0.26
Distance flown	0.10	−0.16	**0.68**	−0.31	0.25	−**0.49**	0.06	−0.32
Conspecific interaction latency	0.25	−0.30	0.13	**0.53**	0.21	**0.33**	−0.01	−0.03
Heterospecific interaction latency	−0.16	−0.12	−0.37	0.20	**0.50**	−**0.60**	−0.13	−0.17
Duration of death−feigning	−0.04	**0.64**	0.49	−0.16	−0.03	**0.37**	−0.02	0.35
Length of time to upturn	−0.04	**0.97**	0.06	0.07	−0.06	0.02	−0.02	0.40
F_appr._	4.20	2.67	2.25	1.98	1.73	1.44	−	−
*p*	<0.001	<0.001	<0.001	<0.001	<0.001	0.001	−	−
Eigenvalue	1.21	0.33	0.22	0.17	0.15	0.10	2.17	0.53

Bold type indicates the main contributors of each axis.

**Figure 3:**
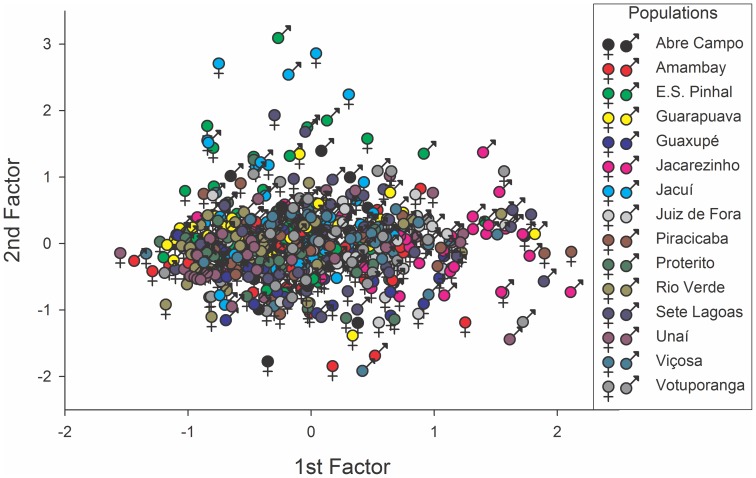
Ordination (principal R-factor) diagram showing the behavioral divergence among adult (male and female) individuals of the maize weevil *Sitophilus zeamais* belonging to 15 populations of this species (**Table 2**).

The multidimensional behavioral constructs representing individual-level weevil ‘personality’ were obtained with the R-factor analysis of the behavioral traits assessed. Such analysis was deemed suitable because the Kaiser’s measure of sampling adequacy was 0.68, significantly exceeding the value of 0.5. Among the factors generated, the first two explained over 97% of the observed variance (Fig. 1). The behavioral traits explaining most of the divergence among individual weevils were the distance walked and walking velocity, with an opposing contribution from resting time (1^st^ factor; Table 3). The 2^nd^ factor exhibited a low Eigenvalue (<1.0) and was retained only aiming the graphical representation (Table 3). The ordination diagram that was obtained with the R-factor analysis representing the diversity of the individual weevil ‘personalities’ is exhibited in Fig. 3. The relative contributions of populations and of individuals within populations for the observed behavioral data variation were estimated to be 7.43 and 92.57%, respectively.

### Insecticide Survival

**Figure 4:**
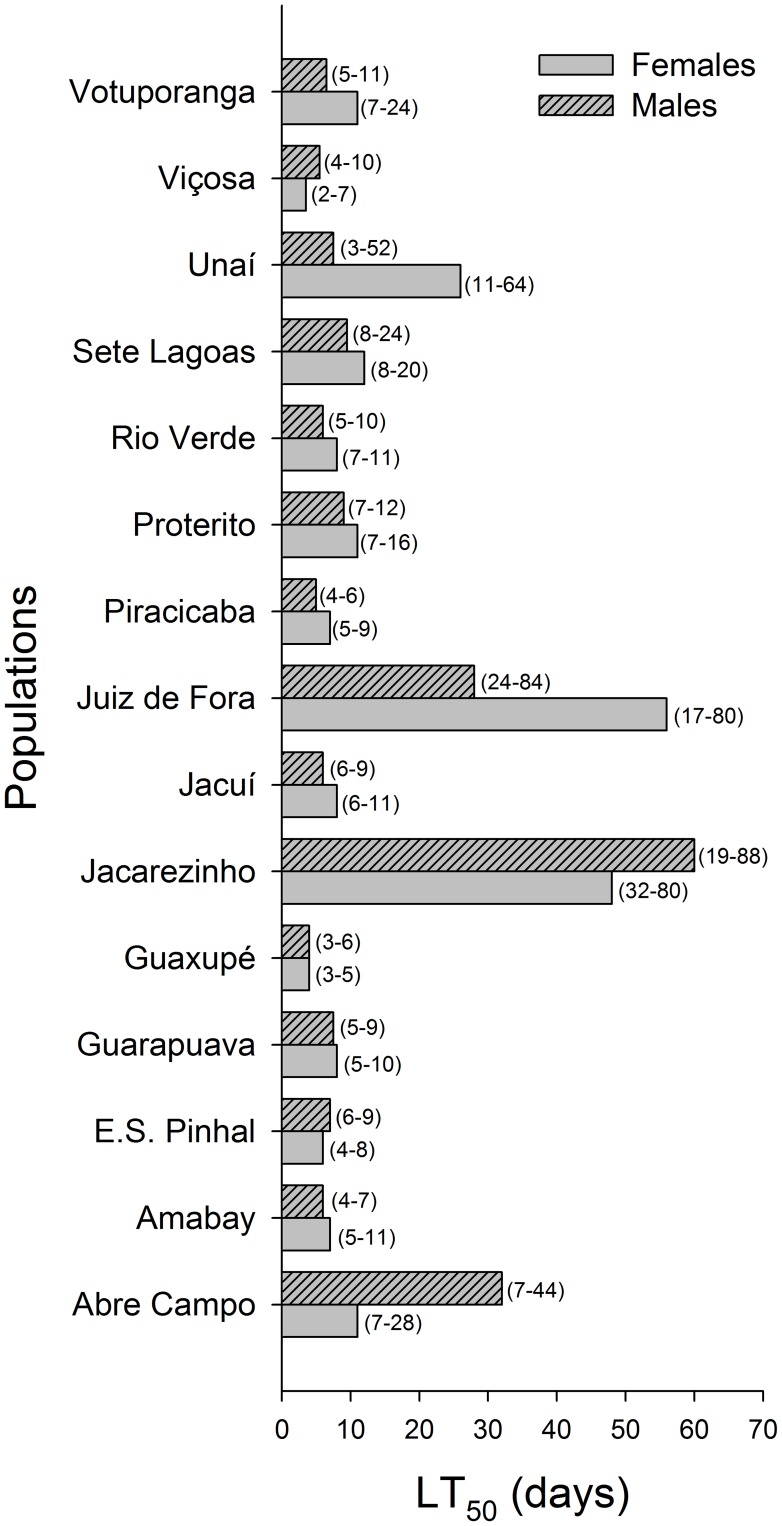
Median survival time (TL_50_) to deltamethrin exposure of both sexes from 15 populations of the maize weevil *Sitophilus zeamais*. The histogram bars indicate the estimated TL_50_’s, and their respective 95% CIs are between parentheses.

The survival analysis of the data from weevils exposed to dried deltamethrin residues on maize kernels indicated significant differences among sexes and populations (Log-rank test, χ^2^ = 223.67, df = 29, *P*<0.001), allowing the estimation of the respective median lethal time (TL_50_’s) using Kaplan-Meyer estimators (Fig. 4). These population estimates were obtained to allow subsequent testing through multiple regression of the potential role of population-based weevil ‘personality’ on the insecticidal efficacy of deltamethrin; this test was not performed for individual weevil ‘personality’, in which the survival time on sprayed kernels was used.

### Weevil ‘Personality’ and Survival to Deltamethrin Exposure

**Figure 5:**
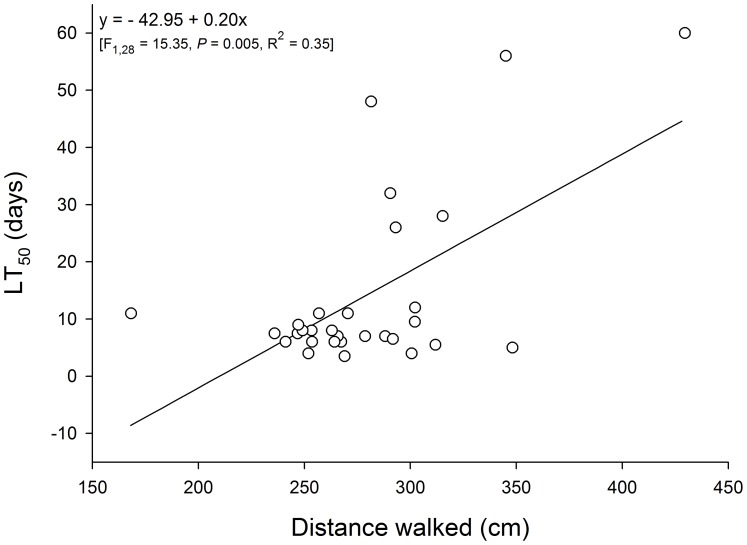
Effect of distance walked on the median lethal time (TL_50_) to deltamethrin exposure of adults from 15 populations of the maize weevil *Sitophilus zeamais*. The symbols indicate the observed data.

**Figure 6:**
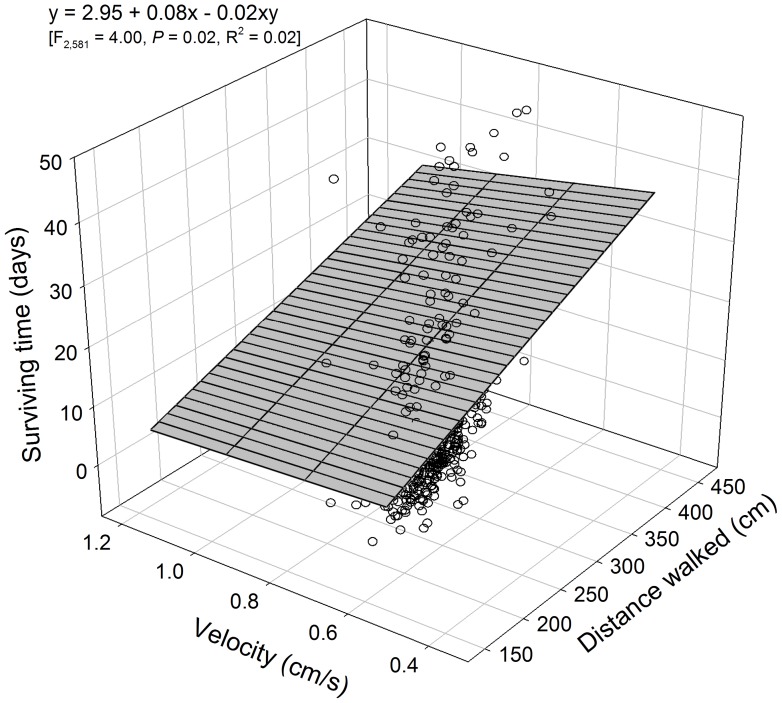
Effect of distance walked and walking velocity on survival time to deltamethrin exposure of adults from 15 populations of the maize weevil *Sitophilus zeamais*. The symbols indicate the observed data.

The model-building procedures used for multiple regression analysis using canonical axes or factors as independent variables to estimate susceptibility to deltamethrin provided neither significant results in the case of factors (*p*<0.05), nor results more robust than when the behavioral traits were directly used in the analysis, which did occur for the canonical axes. Therefore, the behavioral traits were directly used in the multiple regression analysis instead of the multidimensional behavioral constructs obtained with CVA and R-factor analysis. Following this procedure, the distance walked was the only significant predictor of LT_50_ among weevil populations of both sexes, providing robust estimations (R^2^ = 0.35; Fig. 5). When individual behavioral traits were considered to estimate deltamethrin susceptibility, the distance walked was again the main significant estimator of deltamethrin susceptibility, but with a significant contribution of its interaction with walking velocity (Fig. 6). However, the regression model obtained provided only poor estimations of survival (R^2^ = 0.02).

## Discussion

The ‘personality’ concept implies stability (or repeatability) in how the individual behaves, which can be estimated through test-retest correlations. Weevils exhibit significant correlations in behavioral traits subjected to test-retest, indicating that these traits are stable, but their reliability ranged from low to moderate. Low but significant reliability levels (r <0.25) are frequent among animal behavioral traits. In our case, some of the behavioral traits measured for the weevils, such as death-feigning and walking-related traits, reached moderate levels (r ≈ 0.50).

Weevils also exhibited multidimensional behavioral constructs both at the population and individual levels, which are basically diagrammatic representations of ‘personality’ as an individual’s integrated set of behavioral tendencies inferred from multiple empirical measures. Therefore, as observed since the 1970s for vertebrates and the 1990s for invertebrates [65], [66], weevils do exhibit ‘personality’ as defined here, joining the limited group of insects in which ‘personality’ and ‘personality’ variation have been detected [28]–[32]. Previous population-based studies with maize weevils, particularly in interactions with insecticides, previously indicated that some populations are noticeably and consistently more active than others [46], [48], [49], [57], [67]. The present study expands this recognition in maize weevils to the individual level and to multiple behavioral traits encompassing the three behavioral categories (activity, boldness/shyness and exploration/avoidance) explored within the five categories previously proposed (which also includes sociability and aggressiveness) [5]. The personality constructs obtained for weevils suggest that they may also exhibit suites of behavioral correlations across multiple contexts (i.e., behavioral syndromes), particularly between traits within the activity and boldness categories, which deserves future attention.

‘Personality’ is an individual attribute, and here, we observed that the bulk of the variation in the behavioral traits measured were because of individual variation within populations, with only a minor contribution from variation between populations. These results were reflected in the weevil personality constructs generated based on individual variation, which were simpler and more robust than those generated based on population variation, which required more ordination axes with less explicative power than the former. In fact, even the population-based diagram generated with CVA seems to be a rather crude approximation of the individual-based diagram generated with R-factor analysis. However, weevil individual ‘personality’ is roughly translated into weevil population ‘personality’, laying credence to previous population-based studies of weevil behavioral variation [46], [48], [49], [57], [67], [68]. Such a finding also provides support for the potential use of the ‘personality’ concept in comparative studies of populations and even species [3]–[7], [9], [10].

The adaptive value of ‘personality’ is an emerging subject of attention. Among pest insects, the ability to withstand insecticidal applications has both theoretical and practical adaptive importance. Insect behavior is a determinant of insecticide exposure and activity and is also recognized as an insecticide resistance mechanism [38]–[49]. A parallel has been thoroughly explored in psychology, in which child personality has been associated not only with the risk of poisoning but also with injury risk [12], [15], [34]–[37]. By contrast, the potential association between ‘animal personality’ and chemical contamination remains largely unexplored despite its importance, especially for pest insects.

The potential association between maize weevil ‘personality’ as a determinant of its survival to the insecticide deltamethrin was tested in our study. The weevil personality constructs obtained were not associated with survival to deltamethrin exposure, which was better explained by individual behavioral traits, mainly the walking activity, when considering both populations and individuals. Therefore, simple behavioral traits, rather than complex ‘personality’ constructs, were more efficient determinants of survival to deltamethrin exposure, even when physiologically resistant insects were considered although the relationship was weak when individual variation was considered [52]–[55], [68].

We initially expected that increased activity, mainly the walking activity, would be more likely to reduce survival to deltamethrin exposure based on previous studies of the maize weevil [46], [48], [57], [68]. We presented this hypothesis because increased walking activity is expected to increase insecticide exposure and thus favor insecticidal activity. By contrast, our results show that survival to deltamethrin exposure was favored by higher walking activity, which was consistent for both population and individual weevil measurements. However, the general assumption of increased walking activity favoring insecticide exposure does not consider the possibility of escape to untreated surfaces or the possibility of insecticide-mediated behavioral avoidance. Both contingencies occurred in the present study. Only the maize kernels, rather than the vials, received deltamethrin application, allowing the insects to crawl on the vial’s unsprayed inner walls. In addition, deltamethrin is known to frequently induce some behavioral avoidance in populations of the maize weevil [46], [68], [69], which also stimulates their escape to untreated areas, lowering insecticide exposure and explaining the observed association between an increase in the distance walked and an increase in survival to deltamethrin exposure. Furthermore, more active individuals able to cover greater distances are likely of better quality, healthier and thus potentially able to withstand the insecticide effects for longer.

Our experimental set-up was a simplification of field conditions, which may have led to an oversimplified outcome, minimizing the potential role of complex weevil ‘personality’ constructs in survival to deltamethrin exposure. Such confined set-up may also have restrained individual behavioral expression leading to a closer relationship between individual and population results than would take place under unrestrained conditions. Therefore, a more realistic field (or storage) setting for the insecticide survival test may highlight the manifestation of other behavioral traits that could also influence the survival of weevils.

In summary, we report on stable weevil ‘personality’ constructs and highlight the importance of the individual variation of behavioral traits in determining weevil ‘personality’. Nonetheless, the individual weevil ‘personality’ can be roughly translated into weevil population ‘personality’. These relatively complex constructs were not as efficient at determining survival to deltamethrin exposure as the walking activity alone, which plays a significant role in extending survival, and therefore fitness, under insecticide-contaminated conditions.
